# Early transcriptomic response to Fe supply in Fe-deficient tomato plants is strongly influenced by the nature of the chelating agent

**DOI:** 10.1186/s12864-015-2331-5

**Published:** 2016-01-07

**Authors:** Anita Zamboni, Laura Zanin, Nicola Tomasi, Linda Avesani, Roberto Pinton, Zeno Varanini, Stefano Cesco

**Affiliations:** Department of Biotechnology, University of Verona, via delle Grazie 15, 37134 Verona, Italy; Department of Agriculture and Environmental Sciences, University of Udine, via delle Scienze 208, 33100 Udine, Italy; Faculty of Science and Technology, Free University of Bolzano, piazza Università 5, 39100 Bolzano, Italy

## Abstract

**Background:**

It is well known that in the rhizosphere soluble Fe sources available for plants are mainly represented by a mixture of complexes between the micronutrient and organic ligands such as carboxylates and phytosiderophores (PS) released by roots, as well as fractions of humified organic matter. The use by roots of these three natural Fe sources (Fe-citrate, Fe-PS and Fe complexed to water-extractable humic substances, Fe-WEHS) have been already studied at physiological level but the knowledge about the transcriptomic aspects is still lacking.

**Results:**

The ^59^Fe concentration recorded after 24 h in tissues of tomato Fe-deficient plants supplied with ^59^Fe complexed to WEHS reached values about 2 times higher than those measured in response to the supply with Fe-citrate and Fe-PS. However, after 1 h no differences among the three Fe-chelates were observed considering the ^59^Fe concentration and the root Fe(III) reduction activity. A large-scale transcriptional analysis of root tissue after 1 h of Fe supply showed that Fe-WEHS modulated only two transcripts leaving the transcriptome substantially identical to Fe-deficient plants. On the other hand, Fe-citrate and Fe-PS affected 728 and 408 transcripts, respectively, having 289 a similar transcriptional behaviour in response to both Fe sources.

**Conclusions:**

The root transcriptional response to the Fe supply depends on the nature of chelating agents (WEHS, citrate and PS). The supply of Fe-citrate and Fe-PS showed not only a fast back regulation of molecular mechanisms modulated by Fe deficiency but also specific responses due to the uptake of the chelating molecule. Plants fed with Fe-WEHS did not show relevant changes in the root transcriptome with respect to the Fe-deficient plants, indicating that roots did not sense the restored cellular Fe accumulation.

**Electronic supplementary material:**

The online version of this article (doi:10.1186/s12864-015-2331-5) contains supplementary material, which is available to authorized users.

## Background

Iron (Fe) is the micronutrient required in the largest amount by plants and plays a role in key metabolic processes such as respiration, chlorophyll biosynthesis and photosynthesis. This element is a component of the heme group and Fe-sulphur clusters and other binding sites; for its chemical proprieties it is involved in many redox reactions but it can also favour the generation of reactive oxygen species (ROS), which implies a precise control of its uptake, utilization and storage [1].

To counteract the low availability of Fe in soils, higher plants have developed two different strategies for its acquisition from the rhizosphere. The *Strategy I* (all higher plants except grasses) relies on the improvement of Fe solubility through the release of root exudates like protons (*via* an increase of activity of plasma membrane H^+^-ATPase) and organic acids and phenolic compounds followed by a reduction of Fe(III) to the more soluble Fe(II) by a Fe(III)-chelate reductase (FRO) [2]. This reductive step is essential for the acquisition of micronutrient, since Fe(II) is taken up *via* the activity of a divalent cation transporter, Iron-Regulated Transporter (IRT) [1]. *Strategy II* is specific for grasses and is based on the biosynthesis and release of phytosiderophores (PS), which have a strong affinity for Fe(III), and on the uptake of the Fe-PS complexes by a specific transporter, Yellow-Stripe (YS) [1].

Physiological and molecular responses to Fe deficiency in *Strategy I* species have been extensively studied in *Arabidopsis thaliana* [3]. In this model plant, a set of 92 transcripts responsive to Fe deficiency was identified [4]. In tomato roots, a similar number of transcripts (97) was modulated in response to Fe deficiency [5]. More recently, through a co-expression analysis, a group of 180 genes potentially involved in the regulation of *Arabidopsis* responses to Fe shortage was detected [6]. Several works describing plant transcriptional responses to Fe-stress as a comparison between Fe sufficient and Fe deficient condition are present in literature [7–17]. However, no data are available on the modulations taking place during supply after a period of deficiency that is a condition reasonably occurring at the rhizosphere. In the recent years, this matter has been investigated at proteomic level in roots of *Beta vulgaris* [18] and in a *Prunus* hybrid [19], at metabolomic level in roots of *Beta vulgaris* [18], in the xylem sap and leaf extract of *Strategy I* plants [20].

In the rhizosphere the concentration of available Fe depends on the soil pH and on the presence of different types of natural ligands [2, 21–23], such as organic acids [24,25], flavonoids [26, 27], PS [28], microbial siderophores [29] and fractions of the humified organic matter [30, 31]. The acquisition mechanisms of Fe-chelates by *Strategy I* plants is considered to be based on the obligatory step of reduction [23], [32–34] even if recently their possibility to directly absorb Fe-PS has been envisaged [35]. Information about possible differences in the use efficiency of Fe-complexed to natural occurring chelates is still very scarce. It has been reported that fractions of low-molecular-weight water-extractable humic substances (WEHS) complexed with Fe(III) enhanced Fe deficiency responses when compared with natural (citrate) or synthetic [ethylenediaminetetraacetic acid (EDTA)] chelates [36]. Furthermore, a higher amount of ^59^Fe was accumulated in tomato plants treated with Fe-WEHS after 24 h in comparison to other Fe sources [23]. The higher acquisition of Fe from Fe-WEHS was related to a more efficient reduction, rhizosphere acidification and translocation [22, 23, 37].

Here we describe the transcriptional responses of Fe-deficient tomato roots after 1 h of supply with 1 μM Fe chelated to citrate, PS or WEHS. Results showed that the root transcriptional profile of plants supplied with Fe-WEHS is very similar to that of Fe-deficient plants being only two transcripts differentially expressed. The other two natural sources of Fe caused on the other hand a similar modulation of a common set of 289 transcripts. In addition, the Fe-citrate and Fe-PS complexes showed some specific responses as suggested by the modulation of 439 and 119 transcripts after supplying Fe-citrate or Fe-PS alone, respectively.

## Results and discussion

### Iron-(^59^Fe) accumulation from natural Fe-sources by tomato roots

The capability of Fe-deficient tomato plants to utilize different natural Fe-sources was evaluated after 1, 4, or 24 h of treatment performing Fe-uptake experiments and using ^59^Fe complexed with WEHS, citrate or PS. In order to reproduce conditions closer to those where Fe-deficiency symptoms in crops usually appear [38], the uptake medium was buffered at pH 7.5 and each Fe source was used at 1 μM final Fe concentration.

**Fig. 1:**
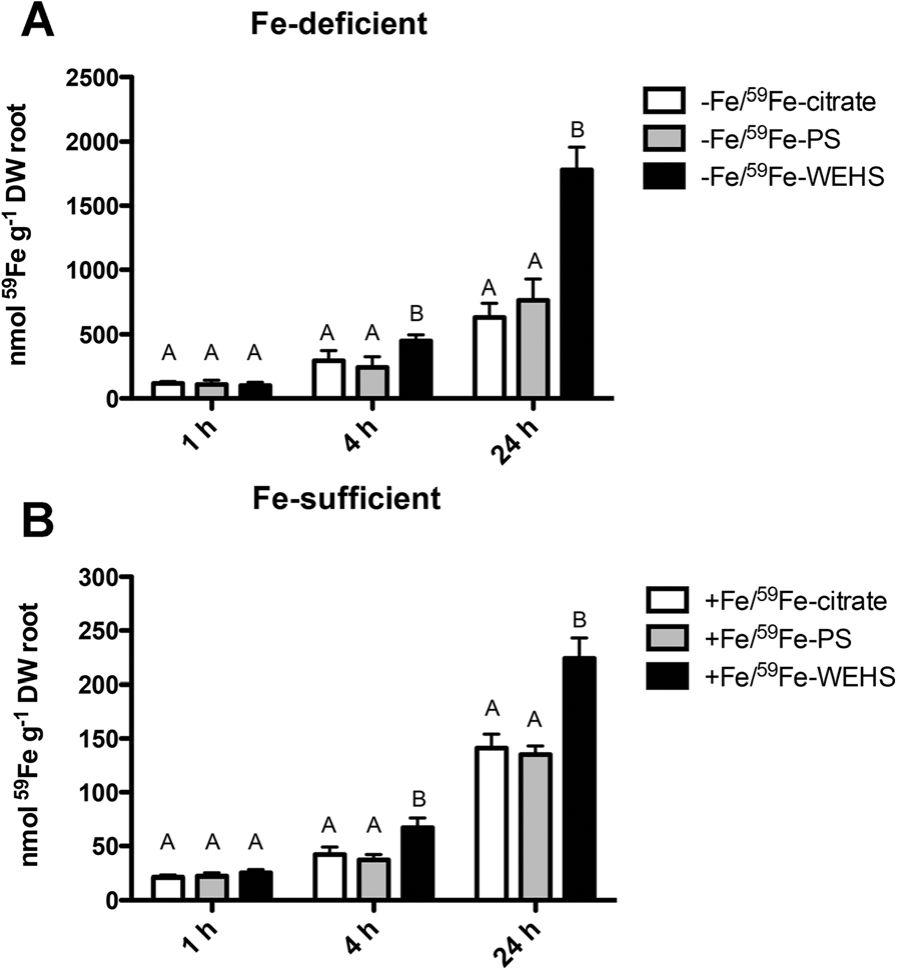
^59^Fe concentration of Fe-deficient and Fe-sufficient tomato plants in response to Fe supply. Fe-deficient (-Fe; **a**) and Fe-sufficient (+Fe; **b**) plants were transferred up to 24 h into a solution (pH 7.5) containing ^59^Fe-citrate, ^59^Fe-PS or ^59^Fe-WEHS at final Fe concentration 1 μM. Data are means ± SD of three independent experiments. Capital letters (**a** to **b**) refer to statistically significant differences (Holm–Sidak method ANOVA, *P* < 0.05)

Fig. 1a shows that after 1 h of supply, the concentration of ^59^Fe accumulated in tomato plants was comparable among all the three Fe treatments exhibiting values around 100 nmol ^59^Fe g^-1^ DW root. Iron content markedly increased up to four folds after 4 h and from six to 18 folds after 24 h. In Fe-WEHS treated plants, the concentration of ^59^Fe taken up was significantly greater than the one measured in plants treated with ^59^Fe-citrate or ^59^Fe-PS at 4 and 24 h. Within each time point, there were no significant differences in Fe content in plants exposed to Fe-citrate and Fe-PS. Fe-sufficient plants (Fig. 1b) showed approximately one order of magnitude lower ^59^Fe accumulation levels than Fe-deficient plants (Fig. 1a) suggesting that responses to Fe shortage are switched off. Also in this case Fe-WEHS treated plants accumulated the highest concentration of ^59^Fe both at 4 and 24 h.

**Fig. 2:**
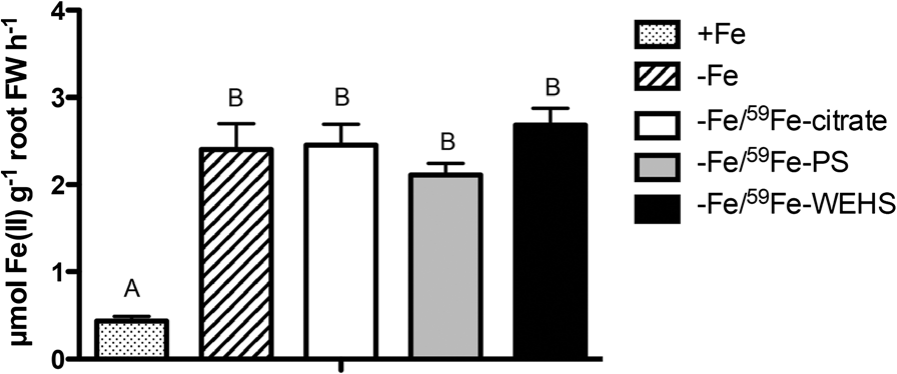
Fe(III)-reduction activity of tomato roots. Fe(III)-reduction activity of intact Fe-deficient tomato plants supplied for 1 h with 1 μM Fe as Fe-citrate, Fe-PS or Fe-WEHS; as control, Fe-deficient plants not treated with any Fe sources (-Fe) or plants treated with 100 μM Fe (+Fe), were also utilized. Data are means ± SD of three independent experiments. Capital letters (a to b) refer to statistically significant differences (Holm–Sidak method ANOVA, *P* < 0.05)

In order to get information on the functionality of Fe-acquisition mechanisms working at the root level of plants supplied with different Fe-complexes, root Fe(III)reduction activity was measured after 1 h of treatment. Fig. 2 reports that the different type of Fe supply did not significantly modify the root Fe(III)-reduction activity.

### Changes in tomato root transcriptome in response to the supply with different natural Fe sources

Root transcriptional profiles of tomato plants in response to 1 h supply with the three different Fe sources were characterized by a genome-wide microarray analysis.

Table 1 reports the numbers of upregulated and downregulated transcripts identified by Linear Models for MicroArray (LIMMA; adjusted p-value ≤ 0.05; |Log_2_(R)| ≥ 1) [39] for each comparison of root transcriptional profiles. To have a further confirmation, the expression level of some differentially expressed transcripts was also tested by Real-time RT-PCR (Additional file 1: Table S1). The number of transcripts differentially expressed in response to Fe-citrate and Fe-PS supply was 728 and 408, respectively. Surprisingly, roots of tomato plants treated with Fe-WEHS showed only two differentially expressed transcripts (one upregulated and one downregulated) in comparison to the Fe-deficiency condition (Table 2) indicating an elevated similarity between these two transcriptional profiles (Additional file 1: Figure S1). This behaviour might explain the higher ^59^Fe content in Fe-WEHS-supplied tomato plants in comparison to Fe-citrate- and Fe-PS-supplied plants after 4 and 24 h (Fig. 1a).

**Table 1:** Number of differentially expressed transcripts resulted by transcriptional profile comparisons of Fe-deficient plants supplied with the three natural sources of Fe and Fe-deficient plants

Comparison	Upregulated transcript	Downregulated transcripts
-Fe/Fe-citrate *vs* -Fe	260	468
-Fe/Fe-PS *vs* -Fe	91	317
-Fe/Fe-WEHS *vs* -Fe	1	1

Differentially expressed transcripts were identified by each transcriptional profile comparison through LIMMA analysis (adjusted p-value ≤ 0.05; |Log_2_(R)| ≥ 1); -Fe: Fe-deficient; -Fe/Fe-citrate, -Fe/Fe-PS or -Fe/Fe-WEHS: Fe-deficient plants supplied for 1 h with Fe citrate, Fe-PS or Fe-WEHS, respectively

**Table 2:** Differentially expressed transcripts cited in the Results and Discussion

#	Probe_ID	Description	p-value, adj; -Fe/Fe-citrate *vs* -Fe	Log_2_(R; -Fe/Fe-citrate *vs* -Fe	p-value, adj; -Fe/Fe-PS *vs* -Fe	Log_2_(R; -Fe/Fe-PS *vs* -Fe	p-value, adj; -Fe/Fe-WEHS *vs* -Fe	Log_2_(R; -Fe/Fe-WEHS *vs* -Fe
*Transcripts similarly affected by Fe-citrate, Fe-PS supply and Fe-WEHS*								
#1	TC215712_723_40_S	R2R3-myb transcription factor, putative	0.001	2.20	0.001	2.14	0.035	2.02
	Transcripts affected by Fe-WEHS supply							
#2	TC194872_1016_38_S	Amino acid transporter, putative					0.035	-1.74
*Transcripts similarly affected by Fe-citrate and Fe-PS supply*								
#3	TC191891_2590_35_S	Plasma membrane H^+^-ATPase	0.003	−1.72	0.010	−1.41		
#4	TC202455_704_34_X2	Fructokinase-2	0.006	−1.39	0.008	−1.47		
#5	TC215677_337_34_X2	Fructose-bisphosphate aldolase	0.008	−1.40	0.016	−1.29		
#6	TC203759_474_40_S	Succinate dehydrogenase	0.001	−1.67	0.003	−1.40		
#7	TC205577_582_35_S	2-Oxoglutarate dehydrogenase, putative	0.002	−1.06	0.002	−1.08		
#8	TC200117_1178_35_S	Methionine synthase	0.004	−1.94	0.009	−1.72		
#9	TC211903_86_41_S	SAM-dependent methyltransferase	0.003	−1.40	0.013	−1.09		
#10	TC212657_260_40_S	SAM-dependent methyltransferase	0.011	−1.10	0.020	−1.06		
#11	TC201480_474_36_S	Phenylalanine ammonia-lyase	0.016	−1.12	0.018	−1.21		
#12	TC207536_637_35_S	ABC transporter family protein	0.002	−1.32	0.003	−1.23		
#13	TC192092_3871_40_S	Cellulose synthase	0.002	−1.34	0.001	−1.60		
#14	TC192418_1233_40_S	Cellulose synthase catalytic subunit	0.002	−1.72	0.003	−1.54		
#15	TC214973_590_40_S	Cellulose synthase A catalytic subunit 3	0.004	−1.11	0.004	−1.23		
#16	TC204385_218_36_X2	UDP-apiose/xylose synthase	0.003	−1.69	0.026	−1.11		
#17	TC192860_1143_39_S	Expansin 1 protein	0.017	1.28	0.026	1.26		
#18	TC198812_683_37_S	Glucan endo-1,3-beta-glucosidase, putative	0.017	1.05	0.010	1.31		
#19	TC201525_569_35_S	Rho GTPase-activating protein At5g61530	0.002	−1.43	0.003	−1.34		
#20	TC196357_464_36_S	ATP/GTP/Ca^++^ binding protein	0.006	−1.60	0.022	−1.29		
#21	TC211495_432_40_S	CBL-interacting protein kinase 1	0.004	−1.18	0.003	−1.45		
#22	TC212764_568_34_X2	Protein IQ-DOMAIN 14	0.003	−1.35	0.008	−1.20		
#23	TC197849_292_41_X2	Ras-related GTP binding protein	0.016	−1.01	0.017	−1.10		
#24	TC207137_449_35_S	RAS superfamily GTP-binding protein-like	0.002	−1.38	0.007	−1.07		
#25	TC196878_2001_40_S	Malic enzyme	0.010	−1.20	0.013	−1.25		
#26	TC191720_1243_40_S	NADH:ubiquinone oxidoreductase-like	0.012	1.29	0.027	1.17		
#27	TC210154_386_41_X4	Glutamate dehydrogenase	0.005	−1.45	0.016	−1.23		
#28	TC192029_938_40_S	Putative basic helix-loop-helix protein bHLH7	0.005	1.17	0.007	1.23		
*Transcript specifically affected by Fe-citrate supply*								
#29	TC208592_1291_35_S	Triosephosphate isomerase, chloroplastic (TIM)	0.004	−1.27				
#30	TC194624_64_34_S	6-Phosphogluconate dehydrogenase	0.002	−1.20				
#31	TC199057_182_40_S	Putative pyruvate dehydrogenase E1 beta subunit	0.036	−1.15				
#32	TC201985_646_40_S	Citrate synthase	0.001	−1.39				
#33	TC212309_491_35_S	Phosphoenolpyruvate carboxylase	0.048	−1.03				
#34	TC193693_30_35_S	NADH dehydrogenase, putative	0.003	−1.21				
#35	TC195215_205_34_X2	NADH dehydrogenase, putative	0.003	−1.41				
#36	TC193283_737_36_S	PHB2	0.001	−1.19				
#37	TC212977_600_37_S	Nitrite reductase	0.049	1.12				
#38	TC196100_60_35_S	Plastid glutamine synthetase GS2	0.004	−1.17				
#39	TC211800_873_40_S	Putative ferredoxin-dependent glutamate synthase 1	0.006	−1.00				
#40	TC197827_1154_40_S	Leucine-rich repeat/extensin	0.030	−1.13				
#41	TC203111_299_41_X2	Extensin-like protein	0.001	−1.34				
#42	TC204863_245_40_S	Extensin-like protein Ext1	0.016	−1.15				
#43	TC216971_395_35_S	Extensin class 1 protein	0.030	1.10				
#44	TC196973_752_38_X2	Pectinesterase	0.019	1.11				
#45	TC210207_490_35_S	Pectinesterase	0.004	−1.18				
#46	TC193792_675_36_S	Putative glutathione S-transferase T5	0.023	1.48				
#47	TC202880_782_35_S	Glutathione S-transferase	0.003	−1.05				
#48	TC207401_351_40_S	Glutathione S-transferase/peroxidase	0.029	1.17				
#49	TC211832_300_41_X2	Glutathione-regulated potassium-efflux system protein kefB, putative	0.034	1.09				
#50	TC197773_1109_35_S	Peroxidase	0.032	1.08				
#51	TC209710_467_35_S	Peroxidase 16, putative	0.012	−1.17				
#52	TC192043_591_40_X3	17.6 kDa class I heat shock protein (Hsp20.0)	0.044	1.03				
#53	TC194246_668_40_S	Heat shock protein 70 (HSP70)	0.002	−1.00				
#54	TC197122_92_35_S	Hsp90 co-chaperone AHA1, putative	0.002	−1.24				
#55	TC207719_568_36_S	Chaperone protein DNAj, putative	0.036	1.06				
#56	TC208736_54_40_S	Chaperonin-60 alpha subunit	0.044	1.05				
#57	TC214617_585_34_X2	Hsp70-interacting protein 1	0.002	−1.17				
#58	TC195735_752_37_S	Avr9/Cf-9 rapidly elicited protein	0.045	1.98				
#59	TC196669_798_35_S	Avr9/Cf-9 rapidly elicited protein 1	0.040	2.57				
#60	TC198633_775_40_S	Avr9/Cf-9 rapidly elicited protein 231	0.019	1.94				
#61	TC200277_609_40_S	Avr9/Cf-9 rapidly elicited protein 194	0.032	1.81				
#62	TC203605_414_40_S	Avr9/Cf-9 rapidly elicited protein 75	0.037	1.59				
#63	TC204489_664_34_X2	Avr9/Cf-9 rapidly elicited protein 20	0.008	2.15				
#64	TC207986_456_38_S	Avr9/Cf-9 rapidly elicited protein 231	0.006	3.69				
#65	TC208735_320_38_S	Avr9/Cf-9 rapidly elicited protein 65	0.006	3.28				
#66	TC200524_503_40_S	WRKY-type DNA binding protein	0.035	1.48				
#67	TC201566_1542_35_S	WRKY-like transcription factor	0.040	1.68				
#68	TC205993_1465_40_S	WRKY transcription factor 1	0.023	2.50				
#69	TC209196_761_40_S	Double WRKY type transfactor	0.014	1.87				
#70	TC214887_802_40_S	WRKY transcription factor-30	0.006	1.20				
#71	TC191592_2431_37_S	GRAS6	0.006	1.09				
#72	TC192009_1993_40_S	GRAS1	0.038	1.14				
#73	TC192616_2450_39_S	GRAS family transcription factor	0.018	1.37				
#74	TC193990_2097_35_S	GRAS9	0.029	1.20				
#75	TC195584_1695_40_S	GRAS4	0.023	1.28				
#76	TC208078_576_35_S	GRAS4	0.007	2.01				
#77	TC213462_831_40_S	GRAS2 transcription factor	0.034	1.93				
	*Transcript specifically affected by Fe-PS supply*							
#78	TC197535_663_40_S	3-Hydroxyacyl-CoA dehyrogenase			0.004	−1.21		
#79	TC203351_729_35_S	Fatty acid desaturase, putative			0.004	−1.08		
#80	TC201677_436_40_S	Acyl-CoA synthetase			0.002	−1.33		
#81	TC195028_1952_40_S	Putative phospholipase C			0.026	−1.12		
#82	TC196917_1568_38_S	Delta(14)-sterol reductase			0.026	1.02		
#83	TC215747_470_40_S	Phosphatidic acid phosphatase			0.041	−1.00		
#84	TC195925_676_35_X2	Ascorbate oxidase			0.049	−1.03		
#85	TC211305_518_35_S	Oligopeptide transporter, putative			0.044	−1.06		
#86	TC196465_645_40_X2	Gibberellin 20 oxidase, putative			0.029	−1.03		
#87	TC204594_438_40_S	TGA10 transcription factor			0.014	−1.26		
#88	TC196692_694_37_X2	GRAS1			0.041	−1.18		
#89	TC211460_599_40_S	bHLH transcription factor JAF13			0.014	−1.01		
#90	TC214149_2_40_S	Myb-like protein			0.029	1.07		
#91	TC204269_546_39_S	Homeobox-leucine zipper protein ATHB-52			0.007	1.12		

Probe ID, description, adjusted *p*-value and Log_2_(R) were reported for each comparison

**Fig. 3:**
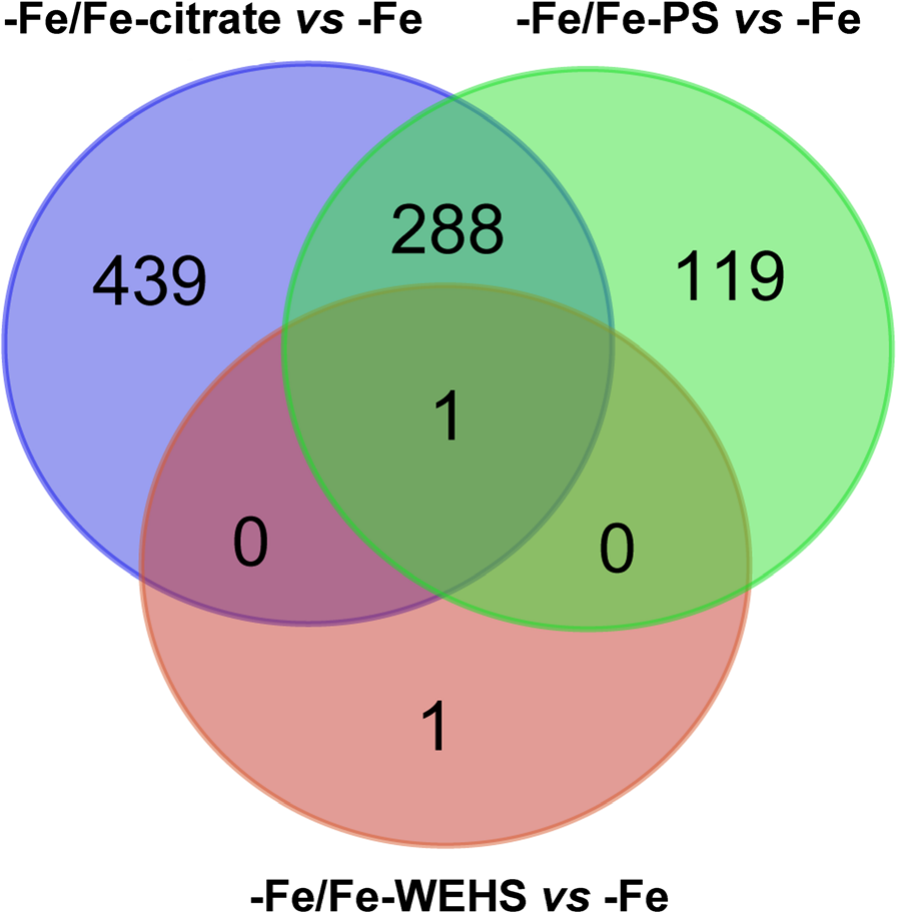
Shared transcripts modulated in response to supply with the three natural Fe sources relative to Fe-deficient plants. Fe-deficient plants were supplied for 1 h with Fe-WEHS (-Fe/Fe-WEHS) or with Fe-PS (-Fe/Fe-PS) or with Fe-citrate (-Fe/Fe-citrate). As control, Fe-deficient plants were used (-Fe)

The upregulated transcript in response to Fe-WEHS-supplied plants had the same behaviour in the plant subjected to the other two treatments (Table 2 and Fig. 3). It encodes a R2R3 MYB transcription factor (#1, Solyc06g005310.2.1; Table 2). The protein encoded by this tomato gene shows highest homology with *Arabidopsis thaliana* MYB48 (35 % of identity) that is not reported to be involved in responses to Fe-deficiency in that plant species. Until now, in *Strategy I* plants, the regulation of Fe-deficiency responses has been described to be controlled by bHLH transcription factors [1, 40]. Data here presented suggest that responses to Fe supply after a period of shortage could be driven by other transcription factors such as this MYB. The sole Fe-WEHS-specific transcript repressed encodes a putative amino acid transporter (#2, Table 2) of unknown function. However, both transcriptional modulations seem unlikely to be responsible for the different efficient use of Fe as Fe-WEHS source.

The root transcriptional profiles of Fe-deficient plants supplied with the three natural sources compared to that of Fe-sufficient plants (LIMMA; adjusted *p*-value ≤ 0.05; |Log_2_(R)| ≥ 1) showed that 90, 1118 and 774 transcripts were after 1 h modulated in response to supply with Fe-WEHS, Fe-citrate and Fe-PS supplies respectively (Additional file 1: Table S2 and Figure S2, Additional file 2: Table S3). The number of differentially expressed transcripts between the Fe-WESH supplied plants and Fe-sufficient ones is similar (90 *vs* 97) to that identified in the previous transcriptional analysis comparing the transcriptome of Fe-sufficient and Fe-deficient roots [5]. It is therefore confirmed that the root transcriptional profile of Fe-deficient plants is very similar to that of plants supplied for 1 h with Fe-WEHS.

### Responses to Fe-citrate and Fe-PS treatments

Differently from the transcriptional behaviour of Fe-WEHS, Fe-citrate and Fe-PS treatments *vs* Fe-deficient determined a modulation of a wider set of transcripts: 289 of them were in common, while 439 (~60 % of total differentially expressed transcripts of -Fe/Fe-citrate *vs* Fe-deficient comparison) and 119 (~ 30 % of total differentially expressed transcripts of -Fe/Fe-PS *vs* Fe-deficient) transcripts were Fe-citrate- and Fe-PS-specific, respectively (Fig. 3). These transcripts are related to specific responses that could be caused by the effect of different chelating agents on root plant metabolism. This hypothesis could be supported by the results obtained comparing the transcriptional profiles of Fe-deficient plant roots supplied for 1 h with Fe (Fe-citrate and Fe-PS) with Fe-sufficient plants (Additional file 1: Figure S2). This analysis revealed that about 52 % and 30 % of differentially expressed transcripts were specific for the -Fe/Fe-citrate *vs* Fe-sufficient and -Fe/Fe-PS *vs* Fe-sufficient comparisons, respectively (Additional file 1: Table S2, Figure S2).

Differentially expressed transcripts in the comparisons -Fe/Fe-citrate *vs* Fe-deficient and -Fe/Fe-PS *vs* Fe-deficient (Table 1), were manually annotated using terms of the biological process of the Gene Ontology (GO) [41] on the basis of BlastP analysis from UniProt database [42] (Additional file 3: Table S4). Eighty-five (11.67 %) and 64 (15.69 %) differentially expressed transcripts in response to Fe-citrate and Fe-PS treatment, respectively, encode proteins without homology with known proteins (“no hits found”). Table 3 shows that “biological process” (transcripts encoding protein involved in “unknown” biological process), “cellular process” and “metabolic process” are the more represented functional categories with similar percentage both for responses to Fe-citrate and Fe-PS supply. The other GO term categories showed similar percentages between the two treatments with the exceptions of “cellular component organization and biogenesis” that is more represented in the response to Fe-citrate relative to Fe-PS (3.53 % *vs* 1.77 %) while “lipid metabolic process” in the response to Fe-PS relative to Fe-citrate (2.00 % *vs* 0.92 %).

**Table 3:** Distribution in main functional categories of transcripts differentially expressed in response to Fe-citrate and Fe-PS supply respectively

-Fe/Fe-citrate *vs* -Fe				-Fe/Fe-PS vs -Fe			
GO Class ID	Definitions	Counts	Fractions	GO Class ID	Definitions	Counts	Fractions
GO:0008150	biological_process	178	27.34 %	GO:0008150	biological_process	122	27.05 %
GO:0009987	cellular process	118	18.13 %	GO:0009987	cellular process	80	17.74 %
GO:0008152	metabolic process	101	15.51 %	GO:0008152	metabolic process	76	16.85 %
GO:0009058	biosynthetic process	33	5.07 %	GO:0009058	biosynthetic process	27	5.99 %
GO:0006139	nucleobase, nucleoside, nucleotide and nucleic acid metabolic process	24	3.69 %	GO:0006139	nucleobase, nucleoside, nucleotide and nucleic acid metabolic process	18	3.99 %
GO:0016043	cellular component organization and biogenesis	23	3.53 %	GO:0019538	protein metabolic process	16	3.55 %
GO:0019538	protein metabolic process	22	3.38 %	GO:0006810	transport	13	2.88 %
GO:0006810	transport	20	3.07 %	GO:0005975	carbohydrate metabolic process	11	2.44 %
GO:0009056	catabolic process	17	2.61 %	GO:0009056	catabolic process	11	2.44 %
GO:0005975	carbohydrate metabolic process	13	2.00 %	GO:0006629	lipid metabolic process	9	2.00 %
GO:0006950	response to stress	12	1.84 %	GO:0016043	cellular component organization and biogenesis	8	1.77 %
GO:0007154	cell communication	9	1.38 %	GO:0007165	signal transduction	6	1.33 %
GO:0006091	generation of precursor metabolites and energy	9	1.38 %	GO:0007154	cell communication	6	1.33 %
GO:0007165	signal transduction	8	1.23 %	GO:0006950	response to stress	6	1.33 %
GO:0006464	protein modification process	8	1.23 %	GO:0006464	protein modification process	6	1.33 %
GO:0006259	DNA metabolic process	6	0.92 %	GO:0006091	generation of precursor metabolites and energy	5	1.11 %
GO:0006629	lipid metabolic process	6	0.92 %	GO:0006259	DNA metabolic process	4	0.89 %
GO:0009628	response to abiotic stimulus	5	0.77 %	GO:0006412	translation	4	0.89 %
GO:0009719	response to endogenous stimulus	5	0.77 %	GO:0015979	photosynthesis	3	0.67 %
GO:0006412	translation	5	0.77 %	GO:0016265	death	2	0.44 %
GO:0015979	photosynthesis	4	0.61 %	GO:0009628	response to abiotic stimulus	2	0.44 %
GO:0016265	death	3	0.46 %	GO:0009607	response to biotic stimulus	2	0.44 %
GO:0008219	cell death	3	0.46 %	GO:0009719	response to endogenous stimulus	2	0.44 %
GO:0000003	reproduction	2	0.31 %	GO:0008219	cell death	2	0.44 %
GO:0009607	response to biotic stimulus	2	0.31 %	GO:0040007	growth	2	0.44 %
GO:0007275	multicellular organismal development	2	0.31 %	GO:0000003	reproduction	1	0.22 %
GO:0040007	growth	2	0.31 %	GO:0009791	post-embryonic development	1	0.22 %
GO:0007049	cell cycle	2	0.31 %	GO:0009605	response to external stimulus	1	0.22 %
GO:0009791	post-embryonic development	1	0.15 %	GO:0009908	flower development	1	0.22 %
GO:0009653	anatomical structure morphogenesis	1	0.15 %	GO:0007275	multicellular organismal development	1	0.22 %
GO:0009605	response to external stimulus	1	0.15 %	GO:0016049	cell growth	1	0.22 %
GO:0009908	flower development	1	0.15 %	GO:0019725	cell homeostasis	1	0.22 %
GO:0019748	secondary metabolic process	1	0.15 %	GO:0007049	cell cycle	1	0.22 %
GO:0016049	cell growth	1	0.15 %	Total		451	100.00 %
GO:0009875	pollen-pistil interaction	1	0.15 %				
GO:0019725	cell homeostasis	1	0.15 %				
GO:0009856	pollination	1	0.15 %				
Total		651	100.00 %				

The distribution in main functional categories on the basis of “biological process” terms was performed using CateGOrizer [79] setting Plant GO slim method and consolidated single occurrences. The analysis was performed using the GO terms of the 643 and 344 transcripts differentially expressed in response to Fe-citrate and Fe-PS respectively and showing homology to “known protein”

### Transcripts commonly modulated by Fe-citrate and Fe-PS supply

The 289 transcripts commonly modulated after Fe-citrate and Fe-PS supply showed the same trend (235 downregulated and 54 upregulated transcripts, Additional file 3: Table S4). Excluding the peculiar behaviour of transcriptome in the presence of Fe-WEHS, this set of transcripts seems to represent the part of transcriptome responsive to the Fe-supply. Twenty upregulated transcripts and thirteen downregulated transcripts did not show any sequence homology with known proteins (“no hits found”). The distribution analysis of the main functional categories of transcripts with homology to known proteins showed that the more abundant terms “biological process”, “cellular process”, “metabolic process” and “biosynthetic process” were similarly represented in both downregulated and upregulated set of transcripts (Table 4). Differences were observed for “transport” with a higher fraction of a downregulated transcript dataset relative to the upregulated one (3.66 % *vs* 1.22 %) while other categories such as “carbohydrate metabolic process”, “catabolic process”, “cellular component organization and biogenesis” and “photosynthesis” were less represented in the downregulated transcript dataset (Table 4).

**Table 4:** Distribution in main functional categories of transcripts modulated both during the Fe-citrate and Fe-PS supply

Upregulated				Downregulated			
GO Class ID	Definitions	Counts	Fractions	GO Class ID	Definitions	Counts	Fractions
GO:0008150	biological process	21	25.61 %	GO:0008150	biological process	91	27.74 %
GO:0009987	cellular process	15	18.29 %	GO:0009987	cellular process	56	17.07 %
GO:0008152	metabolic process	14	17.07 %	GO:0008152	metabolic process	55	16.77 %
GO:0009058	biosynthetic process	5	6.10 %	GO:0009058	biosynthetic process	20	6.10 %
GO:0005975	carbohydrate metabolic process	4	4.88 %	GO:0019538	protein metabolic process	14	4.27 %
GO:0009056	catabolic process	4	4.88 %	GO:0006810	transport	12	3.66 %
GO:0019538	protein metabolic process	3	3.66 %	GO:0006139	nucleobase, nucleoside, nucleotide and nucleic acid metabolic process	12	3.66 %
GO:0016043	cellular component organization and biogenesis	2	2.44 %	GO:0005975	carbohydrate metabolic process	7	2.13 %
GO:0006091	generation of precursor metabolites and energy	2	2.44 %	GO:0009056	catabolic process	7	2.13 %
GO:0006139	nucleobase, nucleoside, nucleotide and nucleic acid metabolic process	2	2.44 %	GO:0016043	cellular component organization and biogenesis	6	1.83 %
GO:0015979	photosynthesis	2	2.44 %	GO:0006950	response to stress	6	1.83 %
GO:0006950	response to stress	1	1.22 %	GO:0006464	protein modification process	5	1.52 %
GO:0006810	transport	1	1.22 %	GO:0006629	lipid metabolic process	5	1.52 %
GO:0007165	signal transduction	1	1.22 %	GO:0006412	translation	4	1.22 %
GO:0007154	cell communication	1	1.22 %	GO:0006259	DNA metabolic process	3	0.91 %
GO:0009719	response to endogenous stimulus	1	1.22 %	GO:0006091	generation of precursor metabolites and energy	3	0.91 %
GO:0006412	translation	1	1.22 %	GO:0016265	death	2	0.61 %
GO:0007049	cell cycle	1	1.22 %	GO:0009628	response to abiotic stimulus	2	0.61 %
GO:0006629	lipid metabolic process	1	1.22 %	GO:0009607	response to biotic stimulus	2	0.61 %
Total		82	100.00 %	GO:0007165	signal transduction	2	0.61 %
				GO:0007154	cell communication	2	0.61 %
				GO:0008219	cell death	2	0.61 %
				GO:0040007	growth	2	0.61 %
				GO:0000003	reproduction	1	0.30 %
				GO:0016049	cell growth	1	0.30 %
				GO:0019725	cell homeostasis	1	0.30 %
				GO:0009791	post-embryonic development	1	0.30 %
				GO:0009605	response to external stimulus	1	0.30 %
				GO:0009908	flower development	1	0.30 %
				GO:0007275	multicellular organismal development	1	0.30 %
				GO:0015979	photosynthesis	1	0.30 %
				Total		328	100.00 %

The distribution in main functional categories on the basis of “biological process” terms was performed using CateGOrizer [79] setting Plant GO slim method and consolidated single occurrences. The analysis was performed using the GO terms of the 41 and 215 transcripts positively and negatively affected respectively in response to both Fe-citrate and Fe-PS and showing homology to “known protein”

The downregulation of a plasma membrane H^+^-ATPase transcript (#3, Table 2) suggested that the acidification of the rhizosphere (component of Fe-acquisition machinery) is more quickly modulated than the expression of transcripts encoding FRO and IRT.

Comparing the modulation of these common supply-specific transcripts with the results of the previous findings in tomato roots [5], we could observe that with the exception of transcripts related to Fe homeostasis (e.g. those encoding FRO, IRT and Natural Resistance-Associated Macrophage Protein, NRAMP) most of the molecular mechanisms involved in the response to the Fe shortage (e.g. glycolysis, TCA cycle, methionine cycles, protein turnover, phenolic compound biosynthesis, root morphological adaptation and signalling) were modulated suggesting the restoration of sufficient nutrient condition. Specifically, we detected a negative modulation of transcripts encoding a phosphofructokinase (PFK; #4), a fructose-bisphosphate aldolase (FBP; #5) for glycolysis and a succinate dehydrogenase (SDH; #6) and a 2′-oxaglutarate dehydrogenase (OGDC; #7) for tri-carboxylic acid (TCA) cycle (Table 2). As far as methionine metabolism and cycle is concerned a methionine synthase (MS; #8) and two S-adenosylmethionine-dependent methyltransferase (SAMT) transcripts (#9 and #10) were repressed (Table 2). Furthermore, transcripts involved in protein turnover such as proteases and peptidases (Additional file 3: Table S4) were mainly negatively affected suggesting the readjusting of the protein metabolism related to the anaplerotic functions. The synthesis and transport of phenolic secondary metabolites appear to be negatively affected as highlighted by the downregulation of transcripts encoding a phenylalanine ammonia-lyase (PAL, #11) and an ATP-binding cassette (ABC) transporter [43] (#12, Table 2). A similar behaviour was observed for transcripts involved in the synthesis of cell wall components (cellulose synthases, CES, #13, #14 and #15 and UDP-apiose/xylose synthase, AXS, #16) while cell wall loosening and modification appeared to be positively influenced by the presence of the micronutrient as highlighted by the upregulation of transcripts encoding an expansin (*LeExp1*, #17, Table 2) and a glucan endo-1,3-beta-glucosidase (#18, Table 2) [44–47]. Concerning the role of Ca^2+^ as secondary messenger during Fe shortage, the negative modulation of signal transduction machinery genes (Rho GTPase-activating protein 1, #19; ATP/GTP/Ca^++^ binding protein, #20; mitochondrial Rho GTPase calcineurin B-like (CBL)-interacting protein kinase 1, #21; Protein IQ-DOMAIN 14, #22; Ras-related GTP binding protein, #23 and #24) is in agreement with the adjustment due to the restored nutrient conditions (Table 2).

Together with the general behaviour described above suggesting the readjustment of metabolic pathways linked to Fe shortage to an adequate nutritional condition, our analysis revealed that other mechanisms are involved in this response.

It is known that the alternative pathway of pyruvate synthesis independent of pyruvate kinase (PK), which is involved in the supply of low-molecular weight organic acid to TCA cycle, is induced under Fe-deficiency [48]; this behaviour would allow to supply reducing power in plants where the functionality of the mitochondrial respiratory chain is limited [49, 50]. A malic enzyme (ME) transcript (#25, Table 2) was repressed in response to Fe supply, hence decreasing the substrate provision to the alternative metabolic cycle. Furthermore, the overexpression of the subunit I of the NADH:ubiquinone oxidoreductase transcript (NADH_UbQ_OxRdtase; #26, Table 2) suggests that Fe supply could restore the respiration chain activity. The repression of a transcript encoding a glutamate dehydrogenase (GDH; #27, Table 2) related to anaplerotic reaction of TCA [51] reinforces the hypothesis of a possible back regulation of TCA cycle during the supply.

Focusing on the transcript involved in molecular processes leading to protein synthesis (*i.e.* translation GO:000641), protein folding (GO:0006457) and protein modification (*i.e.* protein phosphorylation GO:0006468, protein dephosphorylation GO:0006470; protein glycosylation GO:0006486) we observed a downregulation rather than an upregulation (Table 4). This suggests that the new protein synthesis and/or protein modification [52] necessary to respond to the micronutrient depletion are not required in the new restored nutrient condition. We also recorded a downregulation of transcripts involved in DNA and RNA metabolic processes (*i.e.* DNA topological changes GO:0006265; DNA replication initiation GO:0006270; DNA repair GO:0006281; transcription, DNA-templated GO:0006351; regulation of transcription, DNA-templated GO:0006355; RNA splicing GO:0008380) that could be in line with the decrease in protein synthesis. Despite that, other transcripts encoding transcription factors (regulation of transcription, DNA-templated GO:0006355) were upregulated by the treatment with Fe-citrate and Fe-PS (Table 4). Among these transcripts, one encodes for a bHLH (#28) and the other one for a R2R3-MYB transcript (#1). Interestingly this latter transcript is induced by all three Fe-sources (Table 2).

### Transcript specifically affected by Fe-citrate supply

Among the 439 transcripts modulated exclusively by the Fe-citrate treatment, 233 were downregulated and 206 upregulated. Twenty-four downregulated and 26 upregulated transcripts did not show any sequence homology with known proteins (“no hits found”). The distribution analysis of the main functional categories of transcripts with homology to known proteins showed that the more abundant “biological process”, “cellular process” and “metabolic process” were similarly represented both for downregulated and upregulated set of transcripts (Table 5). “Cellular component organization biogenesis”, “biosynthetic process” and “nucleobase, nucleoside, nucleotide and nucleic acid metabolic process” functional categories were more represented in the downregulated transcript dataset while “signal transduction” in the upregulated transcript dataset (Table 5).

**Table 5:** Distribution in main functional categories of transcripts specifically affected by Fe-citrate supply

Upregulated				Downregulated			
GO Class ID	Definitions	Counts	Fractions	GO Class ID	Definitions	Counts	Fractions
GO:0008150	biological_process	59	29.35 %	GO:0008150	biological_process	93	28.53 %
GO:0009987	cellular process	38	18.91 %	GO:0009987	cellular process	59	18.10 %
GO:0008152	metabolic process	31	15.42 %	GO:0008152	metabolic process	48	14.72 %
GO:0019538	protein metabolic process	9	4.48 %	GO:0016043	cellular component organization and biogenesis	17	5.21 %
GO:0006950	response to stress	7	3.48 %	GO:0009058	biosynthetic process	17	5.21 %
GO:0006810	transport	7	3.48 %	GO:0019538	protein metabolic process	15	4.60 %
GO:0009058	biosynthetic process	6	2.99 %	GO:0006810	transport	12	3.68 %
GO:0006139	nucleobase, nucleoside, nucleotide and nucleic acid metabolic process	6	2.99 %	GO:0006139	nucleobase, nucleoside, nucleotide and nucleic acid metabolic process	12	3.68 %
GO:0016043	cellular component organization and biogenesis	5	2.49 %	GO:0009056	catabolic process	9	2.76 %
GO:0007165	signal transduction	4	1.99 %	GO:0006950	response to stress	5	1.53 %
GO:0007154	cell communication	4	1.99 %	GO:0005975	carbohydrate metabolic process	4	1.23 %
GO:0009056	catabolic process	4	1.99 %	GO:0007154	cell communication	4	1.23 %
GO:0009719	response to endogenous stimulus	3	1.49 %	GO:0006464	protein modification process	4	1.23 %
GO:0006464	protein modification process	3	1.49 %	GO:0006412	translation	4	1.23 %
GO:0009628	response to abiotic stimulus	2	1.00 %	GO:0007165	signal transduction	3	0.92 %
GO:0005975	carbohydrate metabolic process	2	1.00 %	GO:0006091	generation of precursor metabolites and energy	3	0.92 %
GO:0015979	photosynthesis	2	1.00 %	GO:0015979	photosynthesis	3	0.92 %
GO:0006629	lipid metabolic process	2	1.00 %	GO:0006259	DNA metabolic process	2	0.61 %
GO:0016265	death	1	0.50 %	GO:0009719	response to endogenous stimulus	2	0.61 %
GO:0006259	DNA metabolic process	1	0.50 %	GO:0006629	lipid metabolic process	2	0.61 %
GO:0019725	cell homeostasis	1	0.50 %	GO:0000003	reproduction	1	0.31 %
GO:0006091	generation of precursor metabolites and energy	1	0.50 %	GO:0009628	response to abiotic stimulus	1	0.31 %
GO:0008219	cell death	1	0.50 %	GO:0009875	pollen-pistil interaction	1	0.31 %
GO:0006412	translation	1	0.50 %	GO:0019725	cell homeostasis	1	0.31 %
GO:0007049	cell cycle	1	0.50 %	GO:0009856	pollination	1	0.31 %
Total		201	100.00 %	GO:0009653	anatomical structure morphogenesis	1	0.31 %
				GO:0007275	multicellular organismal development	1	0.31 %
				GO:0019748	secondary metabolic process	1	0.31 %
				Total		326	100.00 %

The distribution in main functional categories on the basis of “biological process” terms was performed using CateGOrizer [79] setting Plant GO slim method and consolidated single occurrences. The analysis was performed using the GO terms of the 180 and 209 transcripts positively and negatively affected respectively in response to Fe citrate and showing homology to “known protein”

The analysis of downregulated transcripts showed that in addition to those involved in carbohydrate metabolism and TCA cycle above discussed, other genes related to glycolysis (*i.e.* triose-phosphate isomerase, TIM, #29) and pentose phosphate pathway (*i.e.* 6-phosphogluconate dehydrogenase, PGD, #30) were negatively affected (Table 2). A similar behaviour was observed for transcripts of the TCA cycles (pyruvate dehydrogenase E1 beta subunit transcript, PDC, #31; citrate synthase, CS, #32) and of the alternative pathway via PEPC (*i.e.* a transcript encoding PEPC, #33) (Table 2). Two NADH dehydrogenase (NDH) transcripts (#34 and #35) and another one showing homology to the tobacco prohibitin 2 (NbPHB2, #36), which is involved in stress tolerance stabilizing the mitochondrial function [53], were found to be repressed by Fe-citrate treatment. This might be explained as a specific regulation of TCA cycle and mitochondrial activity when Fe is supplied as Fe-citrate, bearing in mind that this organic acid might be absorbed by roots [54].

Other processes related to protein synthesis (“translation”, “translational initiation” and “protein folding”; Table 5) and protein catabolism were mainly repressed (downregulated) in response to Fe-citrate treatment. On the other hand, in the same treatment the functional categories of protein modification processes (e.g. phosphorylation and proteolysis) were similarly represented both for downregulated and upregulated sets of transcripts (Table 5).

The supply with Fe-citrate caused the upregulation of a transcript encoding the Fe-containing enzyme nitrite reductase (NiR, #37, Table 2). This evidence might indicate the restoration of nitrate assimilation, which is known to be altered in Fe-deficient conditions [55]. Besides those involved in protein turnover (see above), other genes putatively related to N recycling were found to be downregulated, such as plastid GS (#38) and ferredoxin-dependent glutamate synthase 1 (GLU, #39).

In addition, Fe-citrate caused the modulation in either directions of transcripts involved in the cell wall metabolism (*i.e.* extensin, EXT, #40, #41, #42 and #43; pectinesterase, PE, #44 and #45), in oxidative stress (#46, #47, #48, #49, #50 and #51) and encoding heat-shock proteins (#52, #53, #54, #55, #56 and #57) (Table 2). These results suggest that the modulation of these processes might be related on one side to the changing of Fe nutritional status, and on the other side to the presence of citrate.

Interestingly, many transcripts involved in the regulation of plant defence response such as *Avr*/*Cf-9* rapidly elicited (ACRE) genes (#58, #59, #60, #61, #62, #63, #64 and #65) and those encoding WRKYs (#66, #67, #68, #69 and #70) were induced by the Fe-citrate supply (Table 2). The activity of these transcripts could be related to the avoidance of Fe toxicity. The involvement of ACRE genes in the response to Al-toxicity in rice roots [56] and the role of a WRKY rice protein in response to excess of Fe [57] has been reported. A similar role in response to Fe toxicity could be ascribed to the upregulation of transcripts belonging to *GAI*, *RGA*, *RCS* (GRAS) gene family (#71, #72, #73, #74, #75, #76 and #77, Table 2). GRAS proteins play a role in the regulation of root growth, nodulation signalling and response to environmental stresses [58]; furthermore, members of this gene family are involved in disease resistance and mechanical stress response in tomato [59].

### Transcript specifically affected by Fe-PS supply

One hundred and nineteen transcripts were specifically modulated in tomato roots by Fe-PS treatment (Fig. 3; Additional file 3: Table S4), 82 and 37 in a negative and in a positive way, respectively. Twenty-one downregulated and ten upregulated transcripts did not show any homology to known proteins (“no hit found”, Additional file 3: Table S4). The distribution in main functional categories highlighted that for the Fe-PS specific transcripts the most abundant categories are “biological process”, “cellular process”, “metabolic process”, “protein metabolic process” and “nucleobase, nucleoside, nucleotide and nucleic acid metabolic process” (Table 6). Transcripts related to “lipid metabolic process” are mainly negatively affected (Table 2) such as those encoding a 3-hydroxyacyl-CoA dehydrogenase (HADH, #78), a fatty acid desaturase (FADS, #79), an acyl-CoA synthetase (ACS, #80) and a phospholipase C (PLC, #81) while only a lipid metabolism-related transcripts, encoding a delta(14)-sterol reductase (Delta-14-SR, #82) was upregulated. In addition to the phospholipase C (PLC) transcript, the treatment with Fe-PS repressed the expression of another transcript encoding a phosphatidic-acid phosphatase (#83, Table 2) suggesting that this Fe source can specifically affect the phospholipid-based signal, which is involved in plant environmental responses [60, 61]. It has been shown that the repression of plant PLCs is related to the response to toxic metals, such as Al^3+^ and Cd^2+^, that implies limiting ROS generation and lipid peroxidation [61, 62]. Iron-PS might negatively affect the phospholipid-based signal that controls responses to Fe, possibly through the reduction of ROS as suggested by the downregulation of an ascorbate oxidase (AO) transcript (#84, Table 2). This AO gene is involved in ascorbic acid biosynthesis in tomato [63] playing an important role as an antioxidant and protecting plant cells during oxidative damage by scavenging free radicals and ROS. On the basis of these results, it might be speculated that Fe is present within the root cells as Fe-PS complex and that this Fe-form could limit Fe-induced ROS production. Indeed there is some evidence that *Strategy I* plants can directly take up Fe-PS complexes [35]. The idea that tomato roots could at least in part adsorb the Fe-PS complexes is supported by the observation that a transcript encoding an oligopeptide transporter “Yellow stripe-like protein” (#85, Solyc03g031920.2.1) was repressed after 1 h of Fe-PS treatment. The rapid back-regulation of this putative Fe-PS transporter would indicate a secondary role in Fe nutrition of tomato plants, while possibly having a role in preventing oxidative damages in the early stages of Fe supply.

**Table 6:** Distribution in main functional categories of transcripts specifically affected by Fe-PS supply

Upregultated				Downregulated			
GO Class ID	Definitions	Counts	Fractions	GO Class ID	Definitions	Counts	Fractions
GO:0008150	biological_process	19	27.94 %	GO:0008150	biological_process	32	29.36 %
GO:0008152	metabolic process	14	20.59 %	GO:0008152	metabolic process	20	18.35 %
GO:0009987	cellular process	11	16.18 %	GO:0009987	cellular process	19	17.43 %
GO:0009058	biosynthetic process	5	7.35 %	GO:0019538	protein metabolic process	6	5.50 %
GO:0019538	protein metabolic process	5	7.35 %	GO:0006139	nucleobase, nucleoside, nucleotide and nucleic acid metabolic process	5	4.59 %
GO:0006139	nucleobase, nucleoside, nucleotide and nucleic acid metabolic process	2	2.94 %	GO:0006629	lipid metabolic process	4	3.67 %
GO:0006464	protein modification process	2	2.94 %	GO:0006810	transport	3	2.75 %
GO:0005975	carbohydrate metabolic process	2	2.94 %	GO:0006464	protein modification process	3	2.75 %
GO:0009056	catabolic process	2	2.94 %	GO:0005975	carbohydrate metabolic process	3	2.75 %
GO:0006810	transport	1	1.47 %	GO:0016043	cellular component organization and biogenesis	2	1.83 %
GO:0007165	signal transduction	1	1.47 %	GO:0007165	signal transduction	2	1.83 %
GO:0007154	cell communication	1	1.47 %	GO:0007154	cell communication	2	1.83 %
GO:0006091	generation of precursor metabolites and energy	1	1.47 %	GO:0006950	response to stress	2	1.83 %
GO:0006412	translation	1	1.47 %	GO:0009058	biosynthetic process	2	1.83 %
GO:0006629	lipid metabolic process	1	1.47 %	GO:0009056	catabolic process	2	1.83 %
Total		68	100.00 %	GO:0006259	DNA metabolic process	1	0.92 %
				GO:0009719	response to endogenous stimulus	1	0.92 %
				Total		109	100.00 %

The distribution in main functional categories on the basis of “biological process” terms was performed using CateGOrizer [79] setting Plant GO slim method and consolidated single occurrences. The analysis was performed using the GO terms of the 27 and 61 transcripts positively and negatively affected respectively in response to Fe-PS and showing homology to “known protein”

A gene encoding the gibberellin 20 oxidase (GA20OX, #86, Table 2), previously hypothesized acting in tomato root morphological changes in response to Fe deficiency [5], was one of those specifically downregulated by Fe-PS supply.

Furthermore, the Fe-PS treatment specifically modulated transcripts encoding transcription factors in a negative (*i.e.* TGA10, #87; GRAS, #88; bHLH JAF13, #89) and in a positive way (Myb-like protein, #90; Homeobox-leucine zipper protein, #91) (Table 2). GRAS transcripts were positively modulated in response to Fe-citrate treatment while, in the case of Fe-PS, one GRAS transcript was downregulated. Our results suggest that some transcription factors could play a role in the response to Fe supply common to different Fe sources, such as the R2R3 MYB transcription factor (#1; Solyc06g005310.2.1, Table 2), while others could be specific for the control of genes and pathways selectively modulated in response to each Fe-source.

## Conclusions

Our results suggest that the root transcriptional response to Fe supply depends on the nature of the ligand (WEHS, citrate and PS). The supply with Fe-WEHS, which has been demonstrated to be able to enhance Fe acquisition responses in *Strategy I* plants [23, 36], did not cause relevant changes in the root transcriptome with respect to the Fe-deficient plants, indicating that roots did not sense the restored cellular Fe accumulation. This result could explain the higher Fe concentration observed after 4 and 24 h in tomato plant tissues supplied with Fe-WEHS as compared to the other Fe-sources. This behaviour is confirmed by a faster and more efficient Fe allocation in the leaf tissue [37]. As a result, Fe-WEHS supply would favour a better distribution of Fe within the plant.

The transcriptional behaviour of tomato roots with the other two natural Fe-sources, Fe-citrate and Fe-PS, underlined that the supply responses are fast and based on a back regulation of molecular mechanisms modulated under Fe deficiency. We also observed some responses specific for each of the two natural Fe sources suggesting a transcriptional response in roots to the molecule used to chelate the micronutrient. Considering transcripts specifically regulated by Fe-citrate, we could hypothesize that citrate is also absorbed by roots causing a further negative regulation of the TCA cycle and influencing mainly cell wall metabolism and the response regulation to stress. Iron-PS specific responses seem to be mainly based on a negative regulation of lipid metabolism and phospholipid-based signal that control ROS responses in the presence of heavy metals.

## Methods

Water extractable humic substances (WEHS) were isolated as reported by Pinton et al. [64] and Fe-WEHS complexes were prepared as described by Cesco et al. [31] by mixing 5 μg organic carbon (Corg) of WEHS fraction for each μmol of FeCl_3_. A thorough chemical characterization of the fractions is described elsewhere [23].

Phytosiderophores (PS) were collected in the root exudate of Fe-deficient barley plants as described by Tomasi et al. [22]. Iron-PS and Fe-citrate were prepared accordingly to von Wirén et al. [65] by mixing an aliquot of Fe-free-PS or citrate (10 % excess of the chelating agent) with FeCl_3_. For radiochemical experiments, ^59^FeCl_3_ was utilized at the specific labeling activity of 144 kBq μmol^−1^ Fe (Perkin Elmer, Monza, Italy).

### Plant material and growth conditions

Tomato seedling (*Solanum lycopersicum* L., cv. ‘Marmande superprecoce’, DOTTO Spa, Italy) were first germinated for 6 days on filter paper moistened with 1 mM CaSO_4_ and consequently grown for other 14 days in a continuously aerated nutrient solution (pH adjusted at 6.0 with 1 M KOH) as reported by Tomasi at al. [22] with 5 μM Fe (Fe-EDTA); thereafter, most of the plants were transferred for a further week to a Fe-free nutrient solution (Fe-deficient) and some tomato plants were transferred for a week to a nutrient solution containing 100 μM Fe-EDTA (Fe-sufficient plants) as control for the Fe(III)-chelate reductase activity. Nutrient solutions were renewed every 3 days. The controlled climatic conditions were the following: day/night photoperiod, 16/8 h; light intensity, 220 μE m^-2^s^-1^; temperature (day/night) 25/20 °C; RH 70 to 80 %.

At the end of the growing period (27 days), Fe-deficient tomato plants clearly showed visible symptoms of Fe deficiency: yellowing of the fully expanded apical leaves, proliferation of lateral roots and root hairs and increase in the diameter of the sub-apical root zone. Twenty-four hours before harvesting, all nutrient solutions were renewed and the pH was adjusted to 7.5 with 10 mM 4-(2-hydroxyethyl)-1 piperazineethanesulfonic acid (HEPES)-KOH. The pH of the growing medium was adjusted to this value to mimic as close as possible the conditions that are occurring in Fe-deficient-inducing soil conditions where plant availability of Fe is reduced. Four hours after the beginning of the light phase, natural Fe-sources (Fe-citrate, Fe-PS or Fe-WEHS) were added to the nutrient solution of Fe-deficient tomato plants to obtain a final concentrations of 1 μM Fe. The same experimental setup was used for radiochemical analyses with ^59^Fe-citrate, ^59^Fe-PS or ^59^Fe-WEHS treatments. The treatment with the three Fe sources lasted up to 24 h; during this period, plant samples were harvested and used for the analyses described below.

For transcriptional analyses, after 1 h (5 h from the beginning of light phase), tomato plants were harvested and collected roots were immediately frozen in liquid nitrogen and stored until further processing at -80 °C. The collection was repeated in three independent cultivations and the roots from six plants were pooled for each treatment. As control, Fe-deficient tomato plants were utilized (without any addition of external Fe sources) prepared in the same experiments and used for the analyses previously presented in Zamboni et al. [5].

### ^59^Fe uptake from natural Fe sources and ferric-chelate reduction capability by roots of intact plants

After 1, 4 or 24 h of treatment with ^59^Fe complexes, plants were transferred to a freshly prepared ^59^Fe-free nutrient solution for 10 min in order to remove the excess of ^59^Fe at the root surface, and then harvested dividing roots and leaves [66]. Root apoplastic ^59^Fe pools were removed using 1.2 g L^−1^ sodium dithionite and 1.5 mM 2,2′-bipyridyl in 1 mM Ca(NO_3_)_2_ under N_2_ bubbling as described by Bienfait et al. [67]; the treatment was repeated three times.

Root and leaf tissues were oven-dried at 80 °C, weighed, ashed at 550 °C and suspended in 1 % (w/v) HCl for ^59^Fe determination by liquid scintillation counting. The ^59^Fe uptake, measured as μg ^59^Fe, is referred to the whole plant (root + leaves) and is presented per g root dry weight .

To determine the root capacity to reduce the Fe(III)-chelates, roots of a single intact (Fe-sufficient or Fe-deficient with or without 1 h supply) tomato plants were incubated in the dark at 25 °C for 60 min in 50 mL of an aerated solution containing CaSO_4_ 0.5 mM, bathophenanthroline disulfonate sodium salt (BPDS) 0.5 mM, 2-(*N*-morpholino)ethanesulfonic acid (MES)-KOH 10 mM (pH 5.5). Thereafter, the absorbance of the solutions was measured at 535 nm at intervals of 15 min and the concentration of Fe(III) reduced calculated by the concentration of the Fe(II)-BPDS_3_ complex formed, using an extinction coefficient of 22.1 mM^−1^ cm^−1^.

### RNA extraction and microarray analyses

Transcriptional analysis was carried out using a Combimatrix chip [68], produced by the Functional Genomics Lab., University of Verona [69]. The chip (TomatoArray2.0) carries 25,789 nonredundant probes (23,282 unique probes and 2507 probes with more than one target) randomly distributed in triplicate across the array, each comprising a 35–40-mer oligonucleotide designed using the program oligoarray 2.1 [70]. The source of sequence information included tentative consensus sequences (TCs) derived from the DFCI Tomato Gene Index [71] Release 12.0 and expressed sequence tags. Eight bacterial oligonucleotide sequences provided by CombiMatrix, 8 probes designed on 8 Ambion spikes and 40 probes based on *Bacillus anthracis*, *Haemophilus ducreyi* and *Alteromonas phage* sequences were used as negative controls. Complete description of the chip is available at the Gene Expression Omnibus [72] under the series entry (GPL13934).

Total RNA was isolated using the SpectrumTM Plant Total RNA kit (Sigma-Aldrich) and quantified by spectrophotometry using NanoDrop™ 1000 (Thermo Scientific). RNA quality was evaluated using Agilent 2100 Bioanalyzer (Agilent). Total RNA (1 μg) was amplified and labelled using the RNA ampULSe kit (Kreatech). After checking the quantity and quality of antisense (aRNA) by spectrophotometry using NanoDrop™ 1000 (Thermo Scientific) and the quality subsequent labelling, 4 μg of labelled aRNA was hybridized to the array according to the manufacturer’s recommendations [68]. Pre-hybridization, hybridization, washing and imaging were performed according to the manufacturer’s protocols. The array was scanned with an Axon GenePix® 4400A scanner (MDS Analytical Technologies).

Analysis of raw data was performed using the open source software of the Bioconductor project [73, 74] with the statistical R programming language [75, 76]. Background adjustment, summarization and quantile normalization were performed using limma package. Probes expressed in all three biological replicates were considered otherwise probes were removed. Differentially expressed probes were identified by linear models analysis [39] using limma package and applying Bayesian correction, adjusted *p*-value of 0.05 and a ⎪FC⎪ ≥ 2. All microarray expression data are available at the Gene Expression Omnibus [72] under the series entry (GSE69419). The data obtained by Fe-deficient and Fe-sufficient plants used in the experiments presented in Zamboni et al. [5] were used as control and submitted with the GEO code: GSE31112. Differentially expressed transcripts between Fe-deficient plants supply for 1 h with the three natural Fe sources and Fe-deficient plants were grouped in main functional categories according to the “biological” terms of the Gene Ontology [30] assigned to each tomato TC or EST (Release 12.0) on the basis of the results of BlastP analysis [77] against the UniProt database [42] (Additional file 3: Table S4). Genes without significant BlastP results were classified as “no hits found” (Evalue < 1e-8; identity > 40 %).

### Real-time RT-PCR experiments

Five hundreds nanograms of total RNA (isolated as previously described) of each sample was retrotranscribed using 1 pmol of Oligo d(T)23VN (New England Biolabs, Beverly, USA) and 10 U M-MulV RNase H^-^ for 1 h at 42 °C (Finnzymes, Helsinki, Finland) following the application protocol of the manufacturers. After RNA digestion with 1 U RNase A (USB, Cleveland, USA) for 1 h at 37 °C, gene expression analyses were performed by adding 0.16 μL of the cDNA to the realtime PCR complete mix, FluoCycle^TM^ sybr green (20 μL final volume; Euroclone, Pero, Italy), in a DNA Engine Opticon Real-Time PCR Detection (Biorad, Hercules, USA). Specific primers (Tm = 58 °C) were designed to generate 80–150 bp PCR products. Three genes were used as housekeeping to normalize the data: LeEF1a, coding for 1-alpha elongation factor (X14449), LeH1, coding for histone protein (AJ224933) and LeUbi3, coding for an ubiquitin protein (X58253). Each Real-Time RT-PCR was performed 4 times on 3 independent experiments; analyses of real-time result were performed using Opticon Monitor 2 software (Biorad, Hercules, USA) and R [74–76], with the qpcR package [78]. Efficiencies of amplification were calculated following the authors’ indications. Sequences of forward and reverse primers and efficiencies were reported in Additional file 1: Table S5 gene.

## Additional files

Additional file 1:
**Figure S1.** Cluster heat map of gene expression data. **Table S1.** Results of Real-time RT-PCR experiments performed for a set of transcripts resulted differentially expressed in the different comparison of microarray analysis. **Table S2.** Number of differentially expressed transcripts resulted by root transcriptional profile comparisons of Fe-deficient plants supplied for 1 h with the three natural sources of Fe and Fe-sufficient plants. **Figure S2.** Shared transcripts modulated in Fe-deficient plants after 1 h in response to supply with the three natural Fe sources relative to Fe-sufficient plants. **Table S5.** Sequence of forward and reverse primers used in Real-time RT-PCR experiments. (PDF 2631 kb) Additional file 2: Table S3. Differentially expressed transcripts resulted by the comparison of root transcriptional profiles of Fe-deficient plants supplied for 1 h with Fe-WEHS, Fe-citrate and Fe-PS with root transcriptional profile of Fe-sufficient plants. Probe ID, adjusted *p*-value and Log_2_(R) were reported for each transcript. (XLS 174 kb) Additional file 3: Table S4. Functional annotation of differentially expressed transcripts resulted by the comparison of root transcriptional profiles of Fe-deficient plants supplied for 1 h with Fe-citrate, Fe-PS and Fe-WEHS with root transcriptional profile of Fe-deficient plants. Probe ID, description, species, identity, score, e-value, Uniprot entry and biological GO term were reported. The adjusted *p*-value and Log_2_(R) were also reported for each transcript. (XLSX 130 kb)
