# Supplementary material for: Early transcriptomic response to Fe supply in Fe-deficient tomato plants is strongly influenced by the nature of the chelating agent
Source: BMC Genomics. 2016 Jan 7;17:35. doi: 10.1186/s12864-015-2331-5 (PMC4705743; doi:10.1186/s12864-015-2331-5)
Supplement: Additional file 1: — Figure S1. Cluster heat map of gene expression data. Table S1. Results of Real-time RT-PCR experiments performed for a set of transcripts resulted differentially expressed in the different comparison of microarray analysis. Table S2. Number of differentially expressed transcripts resulted by root transcriptional profile comparisons of Fe-deficient plants supplied for 1 h with the three natural sources of Fe and Fe-sufficient plants. Figure S2. Shared transcripts modulated in Fe-deficient plants after 1 h in response to supply with the three natural Fe sources relative to Fe-sufficient plants. Table S5. Sequence of forward and reverse primers used in Real-time RT-PCR experiments. (PDF 2631 kb) [file 12864_2015_2331_MOESM1_ESM.pdf]

Figure S1 - Cluster heat map of transcription data.

The hierarchical clustering was generated using Spearman correlation coefficients of expression values. Three biological replicates (I, II and III) were included for each of the following conditions: –Fe: Fe deficient; -Fe/Fe-WEHS: 1 h of supply with Fe-WEHS; -Fe/Fe-PS: 1 h of supply with Fe-PS; -Fe/Fe-CIT: 1 h of supply with Fe-citrate. The color scale indicates the degree of correlation (white, high correlation; red, low correlation).

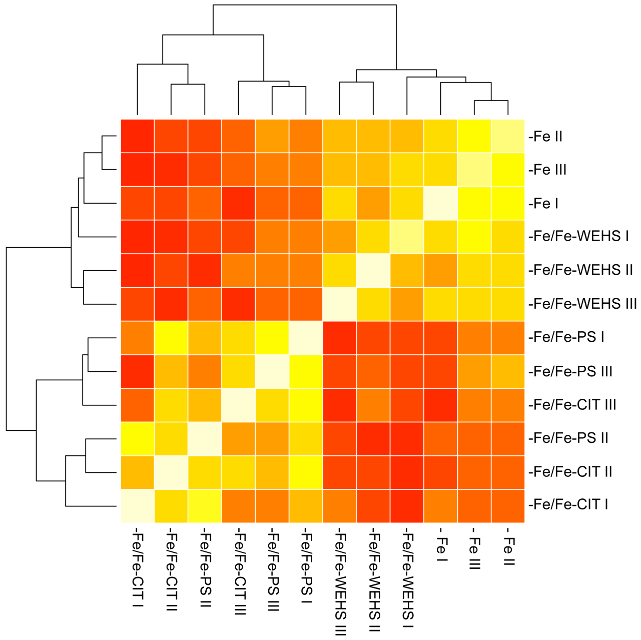

Table S1 - Results of Real-time RT-PCR experiments performed for a set of transcripts differentially expressed in the different comparison of microarray analysis.

Probe ID, description, microarray fold change value and RT-PCR data (means±SE of three biological replicates) were reported.

**Transcripts affected by Fe-WEHS supply**							
**Probe_ID**	**Description**	**Microarray**	**Real-time RT-PCR**				
TC194872_1016_38_S	Amino acid transporter, putative	-3.34	-1.99 ± 0.54				
**Transcripts similarly affected by Fe-citrate, Fe-PS supply and Fe-WEHS**							
		**Fe-citrate *vs* -Fe**		**Fe-PS *vs* -Fe**		**Fe-WEHS *vs* -Fe**	
**Probe_ID**	**Description**	**Microarray**	**Real-time RT-PCR**	**Microarray**	**Real-time RT-PCR**	**Microarray**	**Real-time RT-PCR**
TC215712_723_40_S	R2r3-myb transcription factor	4.60	2.49 ± 0.25	4.41	1.48 ± 0.14	4.06	2.17 ± 0.42
**Transcripts similarly affected by Fe-citrate and Fe-PS supply**							
		**Fe-citrate vs -Fe**		**Fe-PS vs -Fe**			
**Probe_ID**	**Description**	**Microarray**	**Real-time RT-PCR**	**Microarray**	**Real-time RT-PCR**		
NP9287571_278_40_S	TCP protein	-2.17	-2.03 ± 0.01	-2.4	-1.92 ± 0.29		
**Transcripts specifically affected by Fe-citrate supply**							
**Probe_ID**	**Description**	**Microarray**	**Real-time RT-PCR**				
TC203581_620_40_S	BEL1-related homeotic protein 11	-3.61	-2.29 ± 0.17				
**Transcripts specifically affected by Fe-PS supply**							
**Probe_ID**	**Description**	**Microarray**	**Real-time RT-PCR**				
NP000616_1157_38_S	Polygalacturonase 7	2.57	2.36 ± 0.33				
TC196187_747_39_X4	Sucrose synthase	-2.89	-9.97 ± 0.01				
TC199665_1578_35_S	Putative urease accessory protein F	2.64	1.45 ± 0.08				
TC208234_1863_38_S	N-acetyl-glutamate synthase	2.01	1.92 ± 0.03				
TC211460_599_40_S	BHLH transcription factor JAF13	-2.01	-2.60 ± 0.16				
TC216298_625_39_S	Metalloprotease m41 ftsh, putative	2.16	1.70 ± 0.02				

Table S2- Number of differentially expressed transcripts resulted by root transcriptional profile comparisons of plant supplied for 1 h with the three natural sources of Fe and Fe-sufficient plants using LIMMA analysis.

Differentially expressed transcripts were identified by each transcriptional profile comparison through LIMMA analysis (adjusted p-value ≤ 0.05; $|Log_2(R)| \geq 1$); -Fe/Fe plants supplied for 1 h with Fe citrate, Fe-PS or Fe-WEHS; +Fe: Fe-sufficient.

**Comparison**	**Upregulated transcript**	**Downregulated transcripts**
-Fe/Fe-citrate *vs* +Fe	542	576
-Fe/Fe-PS *vs* +Fe	337	437
-Fe/Fe-WEHS *vs* +Fe	68	22

Figure S2- Shared transcripts modulated in Fe-deficient plants after 1 h in response to supply with the three natural Fe sources relative to Fe-sufficient plants.

Fe-deficient plants were supplied for 1 h with Fe-WEHS (-Fe/Fe-WEHS) or with Fe-PS (-Fe/Fe-PS) or with Fe-citrate (-Fe/Fe-citrate). As control, Fe-sufficient plants were used (+Fe).

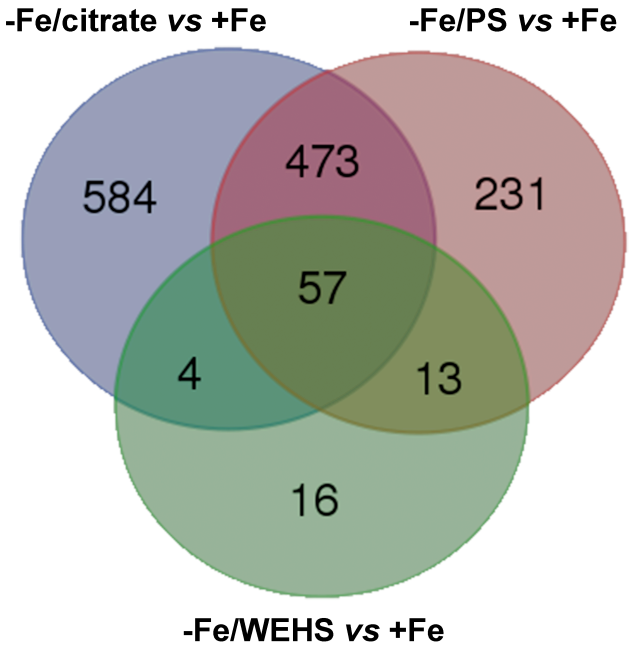

Table S5 - Sequence of forward and reverse primers used in Real-time RT-PCR experiments.

TC_ID (Tentative Consensus ID), description, 5'-3' sequence of forward and reverse primers and efficiency (%) were reported.

TC_ID	Description	Forward primer (5'-3')	Reverse primer (5'-3')	Efficiency
TC192148	Histone protein; LeH1	CAAAGGCCAAAACTGCTACC	AGGCTTTACAGCTGCTTTCG	89.16%
TC196208	Ubiqutin protein; LeUbi3	AGCCAAAGAAGATCAAGCACA	GCCTCTGAACCTTTCCAGTG	87.25%
TC203463	1-Alpha elongation factor; LeEF1a	TGGATATGCTCCAGTGCTTG	TTCCTTACCTGAACGCCTGT	99.15%
NP000616	Polygalacturonase 7	GAGCTTAGGTTGGGAGATGC	GCCTAGCCCATGTTTTGACT	93.15%
NP9287571	TCP protein	GTCTATGGCGGCTGATGATT	TGCGTGGTGTGTTCTTCAAT	60.00%
TC194872	Amino acid transporter, putative	GTCCTGTTGCATCCCTCATT	CGAACAACCATAATGCACGA	90.20%
TC196187	Sucrose synthase	CTCGTGTTCACAGTCTTCGT	TTCAAGATCCCTTTCCCGTG	93.05%
TC199665	Putatative urease accessory protein F	GGATCGGCACTTTTGAGAGT	CATTCAATCCGAGAAGCCCA	95.02%
TC203581	BEL1-related homeotic protein 11	GATGCAATTTCTGGGCAAAT	TTGGCGTAAATGATGATCCA	99.00%
TC208234	N-acetyl-glutamate synthase	CGAGAAGAAGGCATCATCCC	TGAGCTGGTATACGGTCGAT	96.40%
TC211460	bHLH transcritpion factor JAF13	TCACCAAGGAGATGATGCTC	CTCGGCATCTGAATATCGGT	86.00%
TC215712	R2r3-myb transcription factor, putative	AGGCTCACGATGAGAGGAAA	TAGGCGTGAAATCCACATCA	88.60%
TC216298	Metalloprotease m41 ftsh, putative	AATGCAACCTCTTCTCAGCC	ACTTTTGCTTCTTCATGAGGGA	96.35%
